# Increased colonic expression of ACE2 associates with poor prognosis in Crohn’s disease

**DOI:** 10.1038/s41598-021-92979-2

**Published:** 2021-06-29

**Authors:** Takahiko Toyonaga, Kenza C. Araba, Meaghan M. Kennedy, Benjamin P. Keith, Elisabeth A. Wolber, Caroline Beasley, Erin C. Steinbach, Matthew R. Schaner, Animesh Jain, Millie D. Long, Edward L. Barnes, Hans H. Herfarth, Kim L. Isaacs, Jonathan J. Hansen, Muneera R. Kapadia, José Gaston Guillem, Ajay S. Gulati, Praveen Sethupathy, Terrence S. Furey, Camille Ehre, Shehzad Z. Sheikh

**Affiliations:** 1 Center for Gastrointestinal Biology and Disease, University of North Carolina At Chapel Hill, 111 Mason Farm Road, 7312B MBRB, UNC Chapel Hill, Chapel Hill, NC 27599 USA; 2 Department of Genetics, Curriculum in Bioinformatics and Computational Biology, University of North Carolina At Chapel Hill, Chapel Hill, NC USA; 3 Department of Biomedical Sciences, College of Veterinary Medicine, Cornell University, Ithaca, NY USA; 4 Department of Surgery, University of North Carolina At Chapel Hill, Chapel Hill, NC USA; 5 Marsico Lung Institute, University of North Carolina At Chapel Hill, Chapel Hill, NC USA; 6 Department of Gastroenterology and Hepatology, The Jikei University School of Medicine, Tokyo, Japan; 7 Division of Gastroenterology, Department of Pediatrics, University of North Carolina At Chapel Hill, Chapel Hill, NC USA; 8 Department of Biology, University of North Carolina At Chapel Hill, Chapel Hill, NC USA; 9 Division of Rheumatology, Allergy and Immunology, Department of Medicine, University of North Carolina At Chapel Hill, Chapel Hill, NC USA

**Keywords:** Gastroenterology, Crohn's disease

## Abstract

The host receptor for SARS-CoV-2, angiotensin-converting enzyme 2 (ACE2), is highly expressed in small intestine. Our aim was to study colonic ACE2 expression in Crohn's disease (CD) and non-inflammatory bowel disease (non-IBD) controls. We hypothesized that the colonic expression levels of ACE2 impacts CD course. We examined the expression of colonic *ACE2* in 67 adult CD and 14 NIBD control patients using RNA-seq and quantitative (q) RT-PCR. We validated ACE2 protein expression and localization in formalin-fixed, paraffin-embedded matched colon and ileal tissues using immunohistochemistry. The impact of increased *ACE2* expression in CD for the risk of surgery was evaluated by a multivariate regression analysis and a Kaplan–Meier estimator. To provide critical support for the generality of our findings, we analyzed previously published RNA-seq data from two large independent cohorts of CD patients. Colonic *ACE2* expression was significantly higher in a subset of adult CD patients which was defined as the ACE2-high CD subset. IHC in a sampling of ACE2-high CD patients confirmed high ACE2 protein expression in the colon and ileum compared to ACE2-low CD and NIBD patients. Notably, we found that ACE2-high CD patients are significantly more likely to undergo surgery within 5 years of CD diagnosis, and a Cox regression analysis found that high *ACE2* levels is an independent risk factor for surgery (OR 2.17; 95% CI, 1.10–4.26; p = 0.025). Increased intestinal expression of ACE2 is associated with deteriorated clinical outcomes in CD patients. These data point to the need for molecular stratification that can impact CD disease-related outcomes.

## Introduction

Crohn’s disease (CD) is a chronic inflammatory condition of the intestinal tract affecting millions of people worldwide^1–3^. CD patients frequently require immunosuppressant medications, which can increase the risk of infection, particularly for respiratory diseases such as influenza and pneumonia^4^. SARS-CoV-2 infections are increasing world-wide (https://coronavirus.jhu.edu). A significant number of patients present with gastrointestinal symptoms, and high levels of viral RNA in the stool have been detected^5^. This has led the IBD research community to investigate molecules associated with SARS-CoV-2 infectivity with an emphasis on its cognate receptor ACE2^6^. ACE2 is essential for viral entry into epithelial cells and is abundantly expressed in the lung and intestinal epithelium, with markedly higher expression in the small intestine under normal conditions^7^. Expression of two mucosa-specific serine proteases, TMPRSS2 and TMPRSS4, also promote SARS-CoV-2 virus entry into host cells^8^. In the small and large intestine, levels of expression of *ACE2* in patients with CD are dependent on inflammation status and the specific anatomical location^9^.

Disease presentation and progression in CD is highly heterogeneous with regards to location, severity of inflammation, and other phenotypes. Current CD clinical classifications fail to accurately predict disease-related poor outcomes, such as the need for surgery within 5 years of diagnosis^10^. Defining on a molecular basis subset of IBD patients with similar outcomes is essential for developing guidelines for the use of standard IBD therapies. Recently, Suárez-Fariñas, et al., showed high small bowel enterocyte brush border expression of ACE2 and TMPRSS2^6^. In comparison with non-IBD controls, ACE2 expression was decreased and TMPRSS2 expression was increased in the inflamed ileum of CD patients^11,12^. IBD medications, both biologic and non-biologic, did not significantly impact the expression of these genes in the either inflamed or uninflamed small intestine^13^. However, Potdar, et al., revealed that within CD, small bowel ACE2 was reduced in patients subsequently developing complicated disease, and that its expression was restored in responders to biologic therapy^14^. In contrast to the small intestine, increased expression of ACE2 was reported in the inflamed large intestine of CD patients^11,12^. Increased ACE2 expression was restored by non-biologic medications in CD patients and in responders to infliximab^12^. Despite these findings, there remain three major gaps in our knowledge not addressed by recent studies reporting on ACE2 expression and its association with clinical IBD. First, the role of colonic ACE2 in predicting disease course in CD remains unstudied. Second, how the expression of *ACE2* relates to that of other genes, as determined by unbiased transcriptomics, needs to be elucidated in association with the heterogenous disease phenotypes of CD. Verstockt, et al.^11^ utilized bulk and single-cell transcriptomics and performed a global pathway analysis to identify ACE2-related gene regulatory networks. However, their analysis was not driven by clinical phenotypes, and their single cell RNA-sequencing data was derived only from ileum and not from the colon of CD patients. Finally, the relationship of colonic and ileal ACE2 expression in the same patient and its association to disease outcome is unknown.

In this current study, we show that expression of *ACE2*, as well as *TMPRSS2* and *TMPRSS4*, are highly variable in the intestines of adult and pediatric patients with CD, and that their expression levels associate longitudinally with IBD outcome. Our work reveals a novel connection between colonic *ACE2* expression and CD-associated clinical outcomes. These findings motivate future studies that focus on differences in ACE2 regulation between ileum and colon in Crohn’s disease and on whether colonic epithelial SARS-CoV-2 infectivity is greater in the ACE2-high subtype of IBD patients.

## Results

### ACE2 stratifies two distinct molecular subtypes of Crohn’s disease

**Figure 1:**
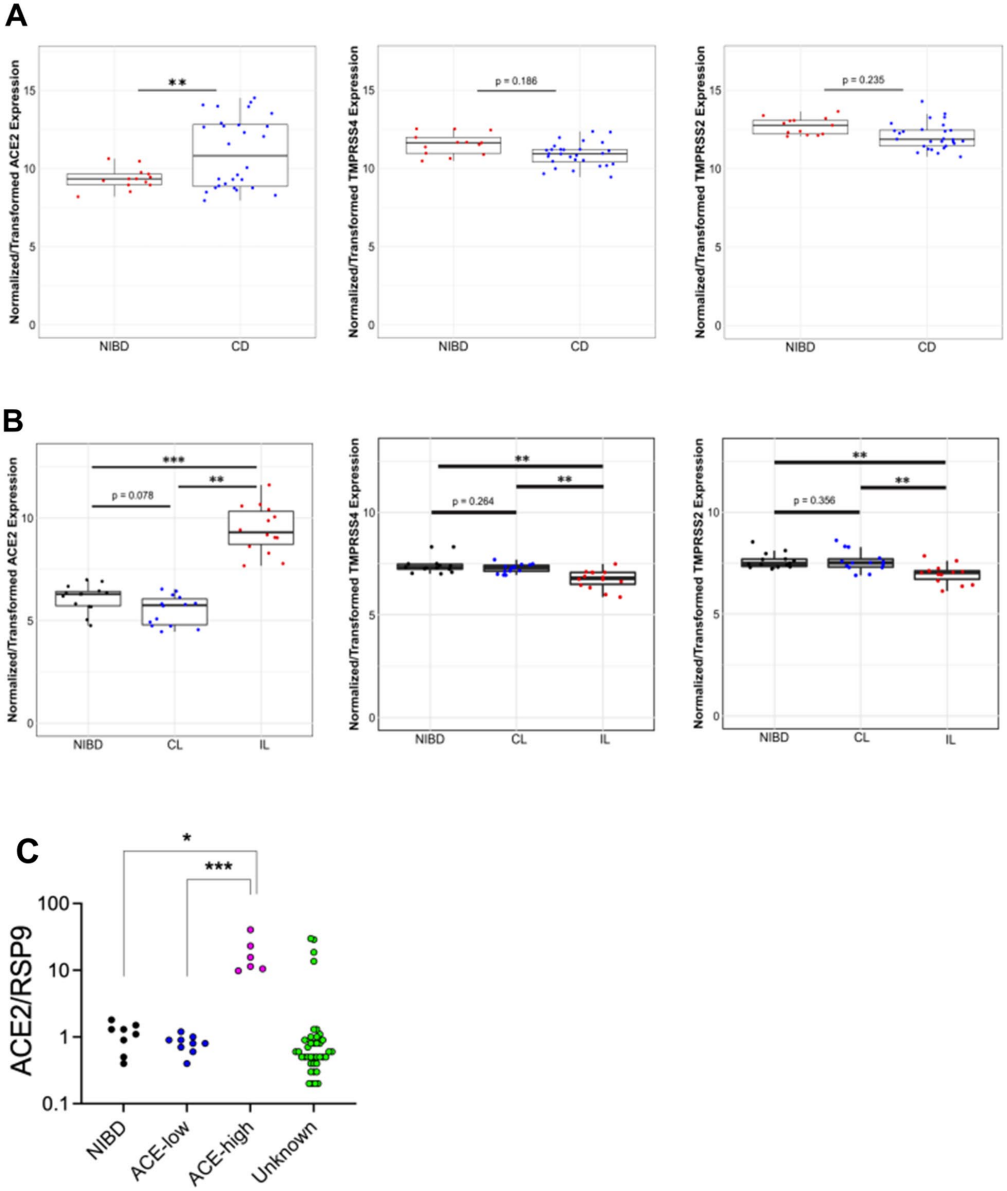
Molecular subtypes of colonic CD. CD patients express *ACE2* at a significantly higher level than non-IBD (NIBD) patients (**A**). Ileum-like CD (IL) patients express *ACE2* and other key marker genes at significantly higher levels than in colon-like CD (CL) patients (**B**). *Adj. P < 0.05; **Adj. P < 0.005; ***Adj. P < 1 × 10^–6^. N = 14 for NIBD and 28 for CD (14 CL and 14 IL). The plots were generated in R v3.6.0 using ggplot2 and prcomp functions. *ACE2* expression was quantified in colonic specimens obtained from 39 additional CD patients (‘Unknown’ ACE2 expression levels) by qPCR and compared with those from 8 NIBD controls, 8 ACE-low CD, and 6 ACE-high CD patients (**C**). *P < 0.05, ***P < 0.001. P-values were determined by Kruskal–Wallis test followed by Dunn’s multiple comparison test. The scatter diagram was created by GraphPad Prism version 9.1.1 (www.graphpad.com).

The clinical presentation and course of CD is highly variable. Previously, we found that gene expression data from non-inflamed colon tissue from adult CD (N = 28) and non-IBD (NIBD) patients (N = 14) clearly segregate CD patients into two disease subtypes (Adult cohort 1)^15^. CD patients in one class largely maintained gene expression profiles of the normal colon (colon-like; CL), whereas in colons of patients in the other class, several normally ileum-specific genes showed robust expression (ileum-like; IL). Altered chromatin accessibility^15^ and microRNA expression^16^ across these classes indicated substantive gene regulatory changes, reflecting a fundamental shift in underlying molecular phenotypes. Interestingly, when considering all CD patients, *ACE2* but not *TMPRSS2* or *TMPRSS4* expression were significantly different between adult CD and non-IBD (NIBD) patients (Fig. 1A). However, when comparing the aforementioned CD subtypes, *ACE2* was elevated and *TMPRSS2* and *TMPRSS4* were decreased significantly in IL CD patients relative to CL CD patients (Fig. 1B). In fact, *ACE2* mRNA levels were 22-fold higher in IL vs CL CD patients. For the purpose of this paper, we will refer to the two CD molecular subtypes here as ACE2-high (IL) and ACE2-low (CL).

To validate these distinct molecular subtypes in CD, we measured colonic *ACE2* expression by qPCR in a second independent cohort of 39 adult CD patients (Adult cohort 2). We stratified them into ACE2-high and ACE2-low groups by comparing against qPCR results from patients from our Adult cohort 1 (n = 6 ACE2-high, 9 ACE2-low). In the new Adult cohort 2, we again identified 4 additional ACE2-high and 35 ACE2-low CD patients (Fig. 1C).

**Figure 2:**
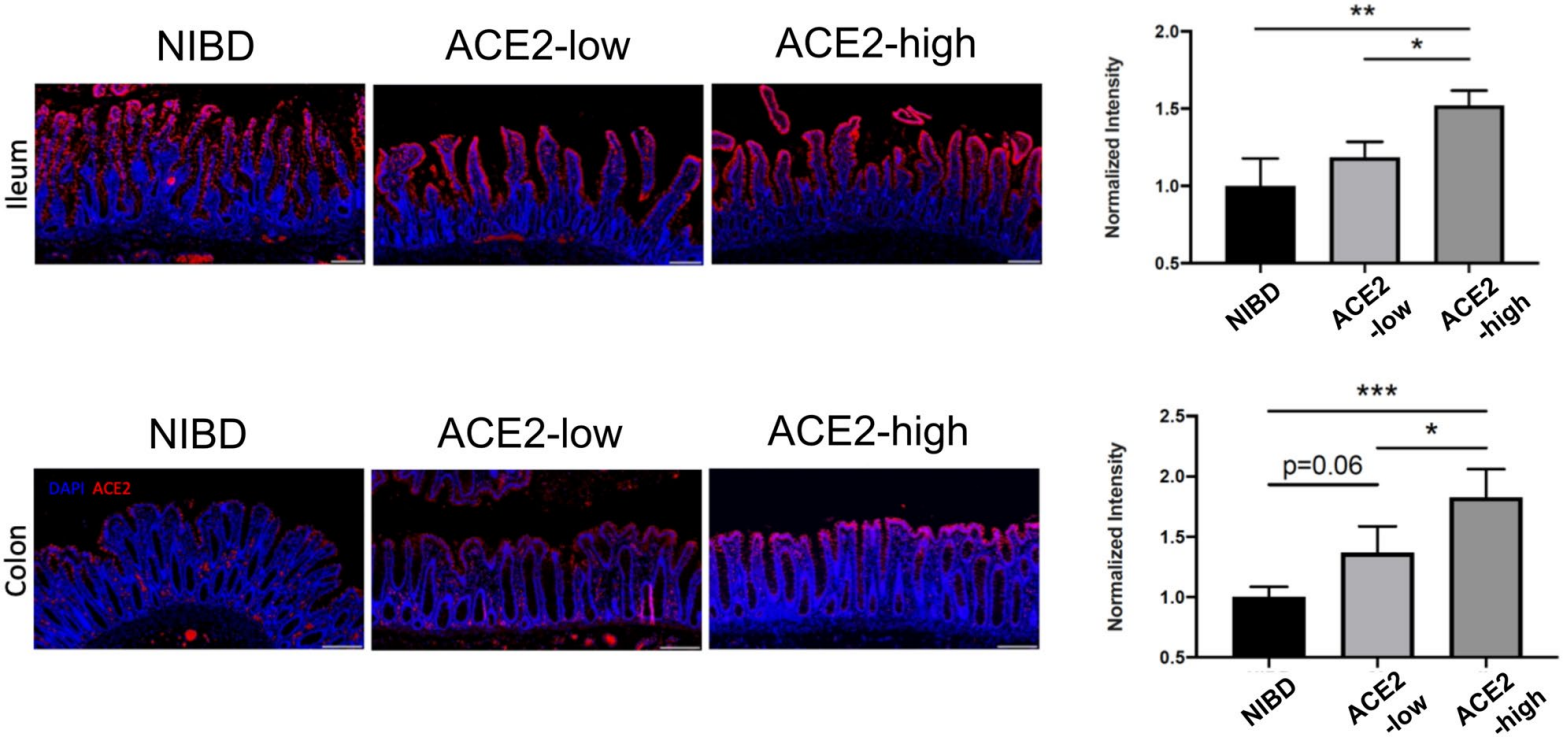
ACE2 IHC reveals increased expression in IL vs CL CD patients. Matched human ileum and colon tissue biopsies from non-IBD (NIBD), ACE-low, and ACE-high patients were stained with anti-ACE2 antibody (pink). Slides were then incubated with DAPI (blue), and ACE2 fluorescent signal intensity was measured using the ImageJ software and normalized to background. N = 4 patients per group. Intensity measurements were averaged per patient and normalized to NIBD group. Significance was determined via one-way ANOVA with multiple comparisons using GraphPad Prism version 9.1.1 (www.graphpad.com). *Adj. P < 0.05, **Adj. P < 0.005, ***Adj. P < 0.001.

Steady state mRNA expression does not necessarily correspond to protein levels, and transcriptomic assays in bulk tissue lack information on tissue localization. To define the expression and localization of ACE2 in CD at high resolution in intact tissue samples, we performed immunohistochemistry (IHC) on matched formalin-fixed, paraffin-embedded (FFPE) uninflamed colon and ileum tissue from 8 CD and 4 NIBD patients. When divided into CD subclasses based on colonic *ACE2* mRNA expression, ACE2-high CD patients exhibited significantly more ACE2 protein signal compared to NIBD and ACE2-low CD patients (Fig. 2). Furthermore, abundant immunoreactivity was displayed in villus enterocytes of ileal tissue with a noted significant difference in ACE2 protein between NIBD and ACE2-high CD patients (Fig. 2). Therefore, we can establish that ACE2 protein levels are consistent with *ACE2* mRNA expression in matched non-inflamed colon and ileal tissue in our patient cohort.

### Colonic ACE2 levels correlate with poor clinical outcomes in Crohn’s disease patients

**Figure 3:**
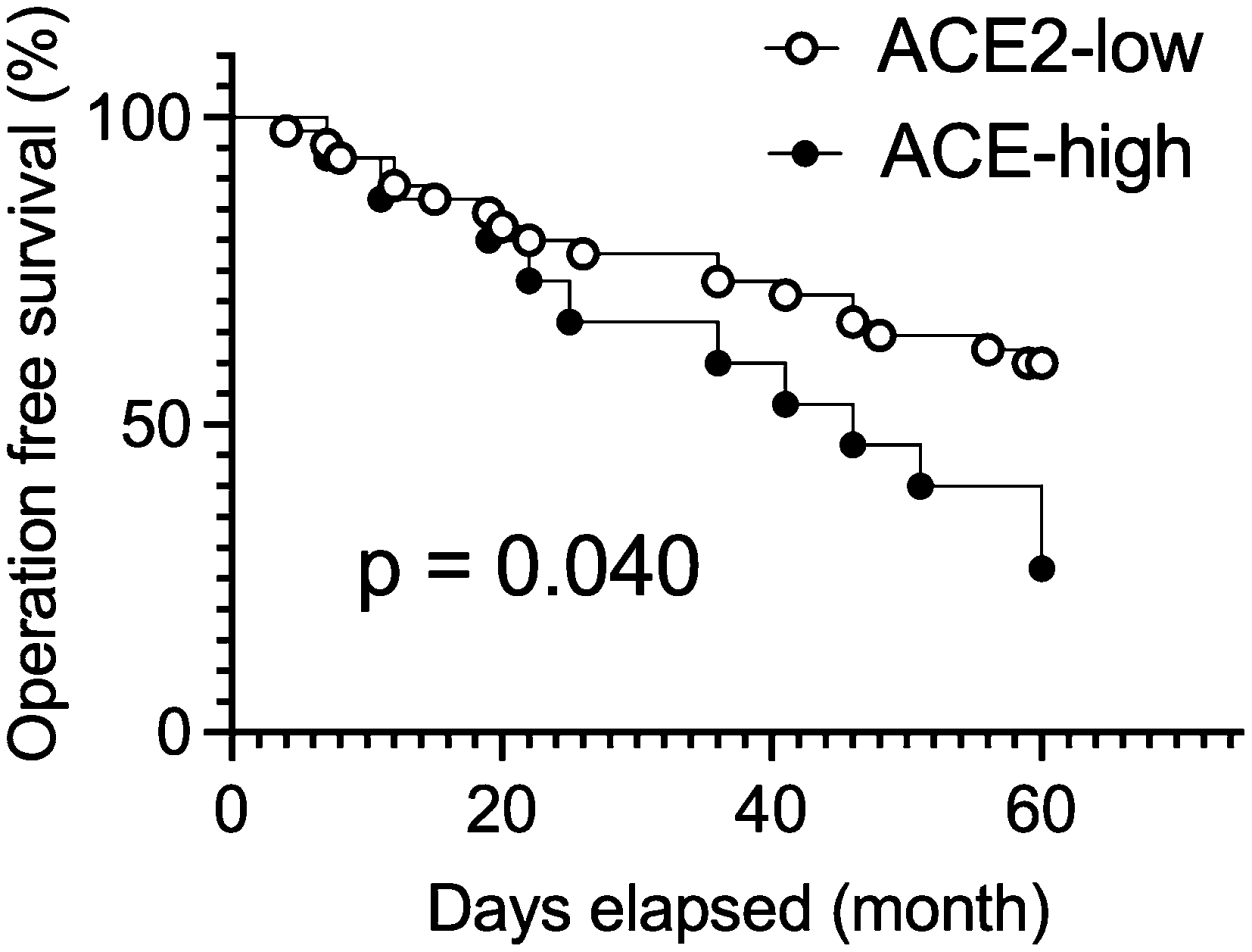
Increased colonic *ACE2* expression is associated with a higher risk of surgery in CD patients. Kaplan–Meier survival analysis for the risk of surgery within 5 years after CD diagnosis in 49 ACE-low and 18 ACE-high CD patients. P-values were determined by a log-rank test using GraphPad Prism software version 9.1.1 (www.graphpad.com).

To determine the clinical impact of colonic *ACE2* expression in CD patients, we compared clinical outcomes between in all CD subtypes (ACE2-low, n = 49; and ACE2-high, n = 18). At the time of CD diagnosis, there was no significant difference in clinical characteristics between the subgroups (Table 1). Notably, though, ACE2-high CD patients showed a significantly higher rate of surgery within 5 years after CD diagnosis than ACE2-low patients (77.8 vs. 44.9%, p = 0.034; Table 2). To better understand this potential relationship to surgery, we generated a Kaplan–Meier plot and performed a subsequent log-rank test that showed a significant difference in the time to first surgery between the two CD subgroups (p = 0.04; Fig. 3). To further elucidate the impact of colonic *ACE2* expression on time to first surgery after CD diagnosis, we next performed Cox regression analysis to account for other variables. Covariates shown in Table 1 and treatment history within 5 years after CD diagnosis (Table 2) were balanced by propensity score for this analysis^11^. We found that being in the ACE2-high CD subclass was a significant independent risk factor for surgery (OR 2.12; 95%CI, 1.10–4.26; p = 0.025; Table 3). Taken together, in this study we discovered that ACE2 expression (mRNA and protein) stratifies two distinct molecular subtypes of CD and that the patients in the ACE2-high subtype have a significantly greater risk of surgery.

**Table 1:** Clinical characteristics at the time of CD diagnosis.

	ACE2-low	ACE2-high	P-value
Number of patients	49	18	
Age (year, median [IQR])	22.0 [16.0, 33.0]	25.5 [18.3, 34.0]	0.301^a^
Gender, Female (%)	35 (71.4)	11 (61.1)	0.610^b^
**Disease location (%)**			0.196^c^
L1	15 (30.6)	6 (33.3)	
L2	12 (24.5)	1 (5.6)	
L3	22 (44.9)	11 (61.1)	
**Disease behaviour (%)**			0.931^c^
B1	8 (16.3)	3 (16.7)	
B2	27 (55.1)	9 (50.0)	
B3	14 (28.6)	6 (33.3)	
L4 disease (%)	7 (14.3)	2 (11.1)	1.000^c^
Perianal disease (%)	15 (30.6)	3 (16.7)	0.356^c^
Current smoking (%)	14 (28.6)	8 (44.4)	0.351^b^

P values were determined by Mann–Whitney test^a^, Chi-squared test^b^, or Fisher’s exact test^c^.

**Table 2:** Treatment history and risk of surgery within 5 years after CD diagnosis.

	ACE2-low	ACE2-high	P-value
Number of patients	49	18	
**Treatment history (%)**			
Systemic steroids	42 (85.7)	13 (72.2)	0.359^a^
Immunomodulators*	36 (73.5)	13 (72.2)	1.000^a^
anti-TNF alpha agents	45 (91.8)	15 (83.3)	0.577^a^
anti-integrin agents	13 (26.5)	2 (11.1)	0.321^b^
anti-IL-12/23p40 agent	6 (12.2)	2 (11.1)	1.000^b^
Surgery (%)	22 (44.9)	14 (77.8)	0.034^a^
**Type of surgery (%)**			
Partial colectomy/enterectomy	16 (32.7)	8 (44.4)	0.545^a^
Ileocecectomy	5 (10.2)	5 (27.8)	0.117^b^
Colostomy/Ileostomy	8 (16.3)	1 (5.6)	0.426^b^

P values were determined by Chi-squared test^a^ or Fisher’s exact test^b^. *Immunomodulators include thiopurines and methotrexate.

**Table 3:** Cox logistic regression analysis for surgery.

Variables	Regression coefficient	SD	P value	OR	95% CI
ACE2-high subclass	0.77	0.35	0.025	2.17	1.10 to 4.26
Propensity Score	3.50	1.13	0.002	33.02	3.62 to 301.30

*SD* standard deviation, *OR* odds ratio, *CI* confidence interval.

### RNA-seq analysis reveals ACE2-high and ACE2-low subclasses in ileal tissue from treatment-naïve pediatric Crohn’s disease patients

**Figure 4:**
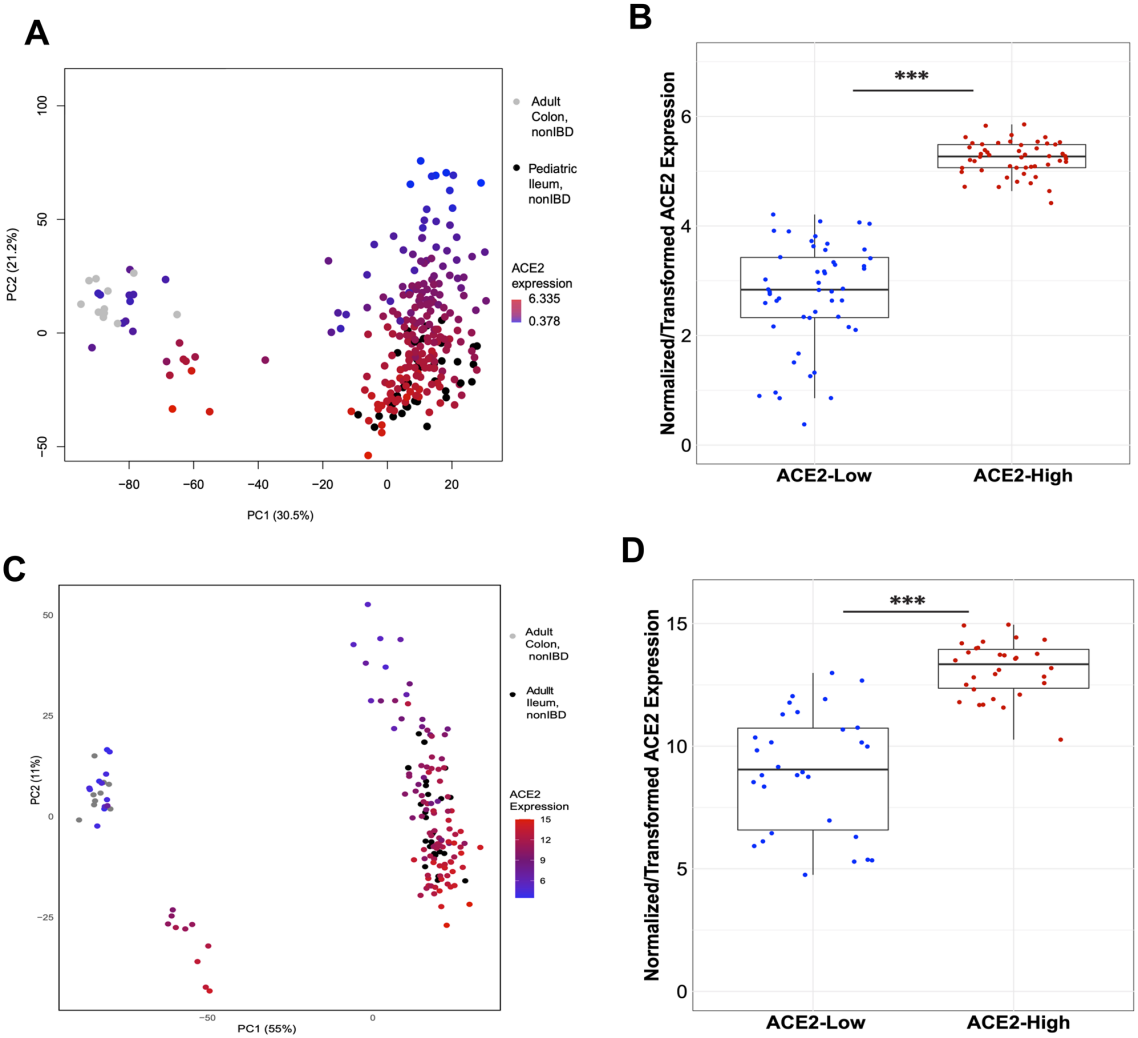
Independent cohorts of adult CD and treatment-naïve pediatric CD ileum samples show similar molecular subtypes. (**A**) PCA of combined RNA-seq data from adult colon tissue and pediatric ileum tissue from CD and NIBD patients replicates ACE2-high and ACE2-low (PC2) subtypes. (**B**) Patients from the two extremes of PC2 (panel A; N = 50, each direction) show significantly different Ileal expression levels of *ACE2* (P < 1 × 10^–6^). (**C**) PCA of combined RNA-seq data from another independent cohort of adult colon tissue and adult ileum tissue from CD and NIBD patients also replicates ACE2-high and ACE2-low (PC2) subtypes. (**D**) Patients from the two extremes of PC2 (panel C; N = 30 each direction) show significantly different Ileal expression levels of *ACE2* (P < 1 × 10^–6^). The plots were generated in R v3.6.0 using ggplot2 and prcomp functions.

*ACE2* expression profiles in adult CD patients may vary due to patient treatment histories. Therefore, we sought to determine whether intestinal tissue from treatment-naïve pediatric CD patients also segregated into similar molecular classes. We used a previously published pediatric RNA-seq dataset from ileal biopsies in age-matched pediatric CD (n = 201) and NIBD (n = 40) patients generated within the Pediatric Risk Stratification Study (RISK)^17^ to compare with the adult colon samples described above. We performed a principal component analysis (PCA) from the expression data (Fig. 4A) and analyzed the RISK samples for *ACE2* expression levels. Unsurprisingly, samples predominantly separated by study (first principal component). However, two molecular subclasses were evident along the second principal component, similar to the first principal component in the single cohort PCAs**.** Further, *ACE2* expression was highly correlated with the second principal component in the pediatric CD samples (Fig. 4B), aligning well with the ACE2-high and ACE2-low subclasses defined by our adult CD colon expression data.

Additionally, we performed a combined PCA using our adult samples with expression data from a second previously published study of adult and pediatric ileal biopsies from NIBD (n = 25, no intestinal inflammation and normal histology) and CD (n = 93) patients^18^ (Fig. 4C). Again, we observed evidence of ACE2-high and ACE2-low subtypes of CD in this independent cohort of patients (Fig. 4D). These data lend strong support toward a generalized stratification of CD subtypes by ileal or colonic *ACE2* expression in both adults and pediatric patients. It is not known whether this stratification is clinically relevant, however.

## Discussion

Angiotensin-converting enzyme 2 (ACE2) has been thrust into the limelight given its role as a receptor for SARS-CoV-2, the virus responsible for the current COVID-19 pandemic. ACE2 is the key effector peptide of the renin-angiotensin system, mediating vasoconstriction and sodium and water retention both directly and indirectly by stimulating aldosterone secretion. While the impact of ACE2 activity on response to infection is still under debate without direct evidence, it is implicated in the response to inflammation and regulation of tissue repair in many organs^19,20^.

A recent single cell (sc) RNA-seq study demonstrated that the ACE2-positive to ACE2-negative cell ratio in the intestinal tract was significantly higher than in the lung^14^. In the lung, co-morbidities dramatically increase alveolar *ACE2* expression and are associated with poor outcomes^21^. Furthermore, location is a critical determinant of intestinal expression of ACE2 (proteinatlas.org). We reveal through generation and analysis of adult colon RNA-seq data and additional analysis with published adult and pediatric ileal RNA-seq datasets in CD patients that expression of *ACE2* defines two molecular phenotypes of CD: the ACE2-low and ACE2-high subsets. Using IHC in matched colon and ileum samples, we validate ACE2 protein expression in apical colonocytes and villus enterocytes for these two patient subsets, as well as NIBD patients.

Our longitudinal analysis from time of CD diagnosis revealed that ACE2-high adult CD patients were associated with increased risk for surgery in the first 5 years after diagnosis. Interestingly, in contrast to worse outcomes in ACE2-high colon expressing adults, we^15^ and others^22^ previously reported worse clinical phenotypes, such as increased risk of macroscopic inflammation with deep ulcers^15^ and the development of disease complication (stricture and penetration)^22^, in a subset of pediatric CD patients with low ileal ACE2 expression compared to those with high ACE2 expression. Although it is not easy to interpret these data from distinct locations (colon and ileum) and populations (adult and pediatric), these regional differences in the clinical impact of *ACE2* expression suggest that active intestinal inflammation alters ACE2 expression, with opposing regulation in ileum and colon^23,24^. There are several points that must be noted with regard to future comparison of our findings with other studies. First, patient selection is critical as is tissue of origin for analysis, including the inflammatory state of the tissue, which can impact gene expression and interpretation of the results^13^. Second, *ACE2* expression in the intestine increases with age, making it important to critically evaluate its role separately in different age groups^13^. Finally, while differences between IBD and NIBD are important, our molecular stratification of CD patients allows for the investigation of two distinct molecular subtypes linked to different clinical phenotypes.

In the small intestine, inflammation and the specific anatomical location were also shown to influence expression of *ACE2* in patients with IBD^9^. We and others showed that in all intestinal segments, *ACE2* expression is much higher in intestinal epithelial cells (IECs) compared to other cells types^25^ (proteinatlas.org). Therefore, understanding how variation in *ACE2* expression and function in IECs impacts disease activity and COVID-19 severity in IBD patients is critical for managing these individuals. Data from the ongoing SECURE-IBD registry (covidibd.org) indicates unsurprisingly that corticosteroid use increases the risk of severe COVID-19 outcomes > fivefold in IBD patients. Recently, Lukin, et al., showed within an inpatient IBD cohort that severe sequelae of COVID-19 were lower than in matched non-IBD controls, suggesting a protective effect of IBD^26^. It remains to be seen if variable colonic or ileal *ACE2* expression in response to IBD therapeutics is responsible for these observations.

There are several limitations in our study. First, the sample number was small. Our findings are the first to report colonic *ACE2* expression with poor outcomes in clinical CD. Future studies incorporating a larger number of patients from a single cohort will help validate our findings. Second, like recently published studies looking at ileal ACE2^12,22,27^, our clinical analysis is retrospective. The clinical impact of colonic and ileal *ACE2* expression should be prospectively validated in future studies. Finally, we did not examine the biological role of ACE2 in IEC homeostasis of CD patients. The biological mechanisms impacted by ACE2 in the intestine remain largely unknown and warrant further study. ACE2 functions in the renin-angiotensin system (RAS), counterbalancing the deleterious effects of angiotensin II on the cardiovascular system^28^. Intestinal ACE2 is a chaperone for the amino acid transporter B^0^AT1, a complex in IECs which regulates the gut microbiota^28^. Gut microbiota composition and function, particularly the presence and activity of bacterial and viral pathogens, greatly influence local and systemic immune responses in IBD^29^. Mechanisms driving expression of *ACE2* and its co-receptor *TMPRSS2* remain unclear. Using an elegant epigenetic approach coupled with genetically manipulated murine models, Chen, et al., found CDX2, HNF4, SMAD4 and GATA transcription factors bind near Ace2 and Tmprss2 resulting in altered chromatin looping and epigenetic modifications with significant impact on *ACE2* and *TMPRSS2* gene expression^25^.

Regardless of these limitations, our present study is significant because we show colonic *ACE2* expression is a potential prognostic biomarker in CD. Given its well-described link to COVID-19 outcomes in the lung, it is plausible that ACE2 may also serve as a possible injury outcome measure for COVID-19 in patients with IBD. The implications of molecular stratification of CD patients can lead to rapid modification of current therapy in IBD patients impacting the natural course of disease. While actual evidence is still scarce, it is hoped that further understanding of the role of ACE2 in IBD pathology and therapeutic responses will ground its use as a biomarker of disease activity and treatment responses contributing to the refinement and development of new therapeutic strategies.

## Materials and methods

### Subjects, samples, and clinical information

#### Adult cohort 1

Colonic mucosa was obtained from surgically resected colon specimens from patients with an established diagnosis of CD and NIBD controls between February 2012 and Jan 2018^15^. All samples were collected from disease-unaffected regions without macroscopic inflammation and were from ascending colon. Clinical information was retrospectively collected from medical records up to 5 years after CD diagnosis.

#### Adult cohort 2

Colonic mucosa was obtained from endoscopically taken biopsy specimens from patients with an established diagnosis of CD between April 2012 and November 2019. All biopsy samples were collected from disease-unaffected regions without macroscopic inflammation and were from ascending colon. Clinical information was retrospectively collected from medical records up to 5 years after CD diagnosis.

#### RISK cohort

Gene expression profiles was studied in treatment-naïve pediatric CD patients using RNA-Seq data from GSE57945 which includes endoscopically-taken ileal biopsies^17^.

In the Adult cohorts, the inclusion and exclusion criteria are as following.

### Inclusionsec criteria

1. Adults who are scheduled to receive surgical intervention for treatment of their documented CD or non-IBD disease (any condition that is an indication for colonic resection as shown in Supplementary Table) (Adult cohort 1), or Adults who are scheduled to receive colonoscopies for their documented CD (Adult cohort 2).
2. Adult subjects of any sex or race, ages 18–99.
3. Able to understand and read English.

### Exclusion criteria

1. Any patient with a bloodborne disease over University of North Carolina BSL2 capabilities, meaning any person who has or was potentially exposed to bloodborne infectious diseases (e.g., HIV or bloodborne hepatitis).
2. Patients who are incarcerated.
3. Patients who are pregnant or breastfeeding.
4. Patients who are taking anticoagulants and/or have bleeding disorders precluding safe collection of samples.

### RNA isolation, sequencing, and processing

Adult samples from UNC hospitals were isolated and sequenced as previously described^15^. Briefly, RNA was isolated using the Qiagen RNeasy Mini Kit following the manufacturer’s protocol, and RNA purity was assessed with Thermo Scientific NanoDrop 2000. RNA-seq libraries were prepared using the Illumina TruSeq polyA + Sample Prep Kit. Paired-end (50 bp) sequencing was performed on the Illumina HiSeq 2500 and 4000 platforms. The obtained data was downloaded from GEO (accession number GSE137344).

Cutadapt v2.9 (https://doi.org/10. 14806/ej.17.1.200) was used to remove sequencing adapters and filter low quality reads (-q 10). Quantification of sequencing reads was performed using Salmon v1.2.^30^ to the hg38 genome with GC-bias and sequence-specific parameters enabled (–gcbias and –seqbias, respectively), and tximport v1.12.3 (10. 12688/f1000research.7563.1) was used to summarize transcript-level to gene-level abundance estimates using R v3.6.0. Pediatric CD samples were processed as described previously^12^ and downloaded from GEO (accession number GSE57945).

### Data availability statement

The sequencing data underlying this article are available in public sequencing data from GEO, sratoolkit v2.10.1 (http://ncbi.github.io/sra-tools/). The remaining data underlying this article are available in the article and in its online supplementary material.

### RNA analysis

Raw sequencing counts from Salmon were DESeq2 normalized and VST transformed^31^. Box plots were generated using ggplot2, and PCA was performed using the prcomp function in R v3.6.0 (citation: R Core Team (2020). R: A language and environment for statistical computing. R Foundation for Statistical Computing, Vienna, Austria. https://www.R-project.org/).

### Immunohistochemistry (IHC)

Formalin-fixed, paraffin-embedded (FFPE) uninflamed colon and ileum tissue matching with the RNA-seq samples were available in 8 CD and 4 NIBD patients. The ileum and colon tissue biopsies from these patients were fixed in 10% (vol/vol) neutral buffered formalin, embedded in paraffin, and prepared as histological sections. After deparaffinization and epitope retrieval in 1X citrate buffer solution, sections were blocked for 1 h in 3% BSA before immunostaining was performed. Polyclonal goat anti-ACE2 antibody (R&D Systems #AF933) was applied overnight at 4 °C, followed by a 1 h incubation with a secondary anti-goat (Alexa Fluor 594) antibody the next day. Slides were then incubated with DAPI (Invitrogen #D1306) for 5 min to stain nuclei and mounted using FluorSave Reagent (EMD Millipore #345789). Fluorescence was detected using an Olympus VS120 virtual slide microscope.

### ACE2 signal intensity

ACE2 fluorescent signal intensity was measured using ImageJ software and normalized to background. To facilitate measurements, images of the stained tissue sections were converted to black and white images on the ACE2 channel, removing signal from DAPI. For each section, pixel intensity was measured in three different regions that were selected for optimal histological cut, showing intact villi (ileum) or crypts (colon). Five intensity measurements (e.g., yellow rectangles on supplemental data images) were analyzed per region (Supplemental Fig. 1). N = 4 patients per group. Intensity measurements were averaged per patient and normalized to disease-control group. Significance was determined via one-way ANOVA with multiple comparisons.

### Reverse-transcriptase qPCR analysis

Total RNA was extracted from dissected colonic mucosa, stored in RNAlater using TRIzol reagent and purified with the Total RNA Purification Plus Kit (48300; Norgen Biotek) according to the manufacturer’s instructions. Total RNA was extracted from isolated and cultured colonic IECs using the Single Cell RNA Purification Kit (51800; Norgen Biotek). Complementary DNA for mRNA was generated from 500 ng of RNA using the High-Capacity Complementary DNA Reverse Transcription Kit (4368814; Thermo Fisher Science). Comparative-Ct-TaqMan with a relative quantification qPCR for mRNAs was performed on the QuantStudio 3 RT-PCR system using TaqMan Fast Advanced Master Mix (4444557; Thermo Fisher Science) with individual TaqMan probes (TaqMan Gene Expression assays, assay ID: Hs01085333_m1 [ACE2], Hs02339424_g1 [RPS9]). Expression of *ACE2* was normalized to *RPS9*.

### Statistical analysis

All numeric data in the figures are expressed as means ± standard deviation (SD). Differences between the 2 groups were analyzed by a Mann–Whitney or Fisher exact test. Differences between the 3 groups were analyzed by a Kruskal–Wallis test followed by Dunn’s multiple comparison test. *P* values less than 0.05 were considered significant. The Kaplan–Meier method was used to generate survival curves and differences between 2 groups were evaluated by a log-rank test. GraphPad Prism (version 9.1.1 for macOS; GraphPad Software, San Diego, California, USA, www.graphpad.com) was used for these data analyses. Calculation of propensity score and a Cox regression analysis were performed using R v4.0.3 (citation: R Core Team (2020). R: A language and environment for statistical computing. R Foundation for Statistical Computing, Vienna, Austria. https://www.R-project.org/), and the R package ‘survival’ (citation: Therneau T (2021). A Package for Survival Analysis in R. R package version 3.2–10. https://CRAN.R-project.org/package=survival).

### Ethical considerations

This study was conducted in accordance with the Declaration of Helsinki and Good Clinical Practice. The study protocol was approved by the Institutional Review Board at the University of North Carolina at Chapel Hill (approval numbers: 19-0819 and 17-0236). All participants provided written informed consent before inclusion in the study. All participants were identified by number and not by name or any protected health information.

All authors had access to the study data and reviewed and approved the final manuscript.

## Supplementary Information

Supplementary Information.
