# Supplementary material for: Increased colonic expression of ACE2 associates with poor prognosis in Crohn’s disease
Source: Sci Rep. 2021 Jun 29;11:13533. doi: 10.1038/s41598-021-92979-2 (PMC8241995; doi:10.1038/s41598-021-92979-2)
Supplement: Supplementary file 1 — Supplementary Information. [file 41598_2021_92979_MOESM1_ESM.pdf]

Title: Increased Colonic Expression of ACE2 Associates with Poor Prognosis in Crohn's disease

Authors: Takahiko Toyonaga, MD, PhD [1,7], Kenza C. Araba, BA[2,5], Meaghan M. Kennedy, MS[1,2], Benjamin P. Keith, PhD [1,2], Elisabeth A. Wolber, BA[1], Caroline Beasley, BA[1], Erin C. Steinbach, MD, PhD[1,10], Matthew R. Schaner, BA[1], Animesh Jain, MD[1], Millie D. Long, MD, MPH[1], Edward L. Barnes, MD, MPH[1], Hans H. Herfarth, MD, PhD[1], Kim L. Isaacs, MD, PhD[1], Jonathan J. Hansen, MD, PhD[1], Muneera Kapadia, MD, MME[4], José Gaston Guillem, MD, MPH, MBA[4], Ajay S. Gulati, MD[1,8], Praveen Sethupathy, PhD[3], Terrence S. Furey, PhD[1,2,9], Camille Ehre, PhD[5], Shehzad Z. Sheikh, MD, PhD[1,2]

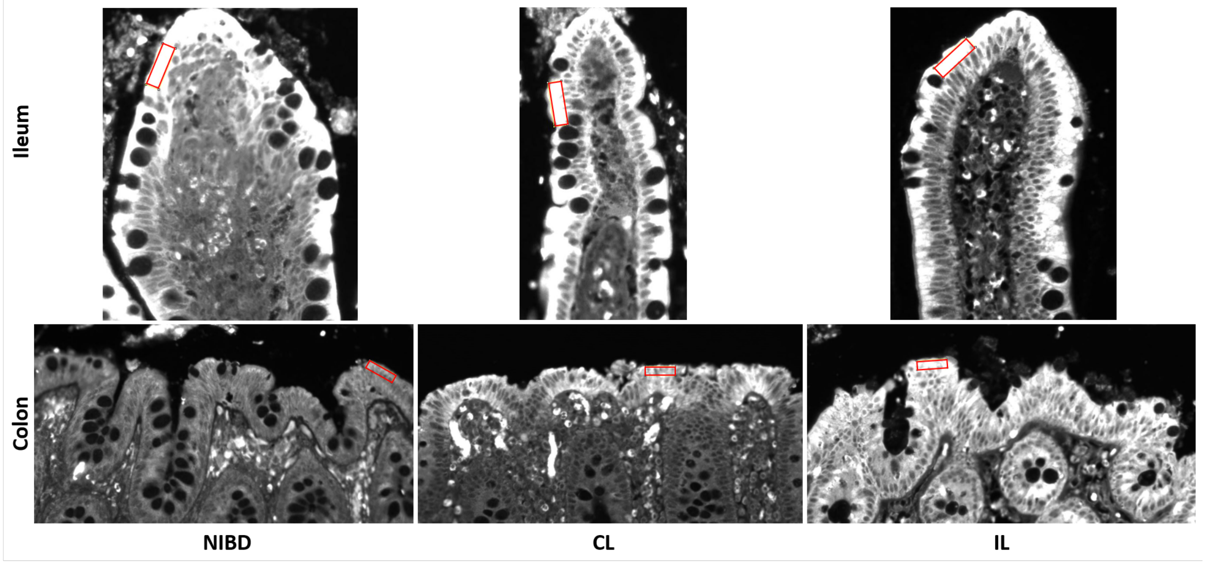

Supplementary Figure 1. ACE2 fluorescent signal intensity was measured using the ImageJ software and normalized to background. Images of the stained tissue sections were converted to black and white images on the ACE2 channel, removing signal from DAPI. For each section, pixel intensity was measured in three different regions that were selected for optimal histological cut, showing intact villi (ileum) or colonocytes (colon). Five intensity measurements (red rectangles) were analyzed per region. N=4 patients per group. Intensity measurements were averaged per patient and normalized to NIBD.

Age at Surgery	Gender	Disease (reason for surgery)
53	Female	colon cancer
52	Male	colon cancer
70	Male	diverticulitis
82	Female	colon cancer
45	Male	colon cancer
44	Male	colon cancer
41	Female	colonic inertia
41	Female	colonic inertia
52	Male	colon cancer
64	Male	colon polyp
45	Female	colonic inertia
70	Male	colon adenoma
62	Male	colon adenoma
49	Female	neuroendocrine tumor

Supplementary Table 1. Clinical characteristics of NIBD patients.
